# A role for the immune system in advanced laryngeal cancer

**DOI:** 10.1038/s41598-020-73747-0

**Published:** 2020-10-27

**Authors:** Marta Tagliabue, Fausto Maffini, Caterina Fumagalli, Sara Gandini, Daniela Lepanto, Federica Corso, Salvatore Cacciola, Alberto Ranghiero, Alessandra Rappa, Davide Vacirca, Maria Cossu Rocca, Daniela Alterio, Elena Guerini Rocco, Augusto Cattaneo, Francesco Chu, Stefano Zorzi, Giuseppe Curigliano, Susanna Chiocca, Massimo Barberis, Giuseppe Viale, Mohssen Ansarin

**Affiliations:** 1 Division of Otolaryngology and Head and Neck Surgery, IEO, European Institute of Oncology, IRCCS, Via Ripamonti 435, 20141 Milan, Italy; 2 Division of Pathology, IEO, European Institute of Oncology IRCCS, Via Ripamonti 435, 20141 Milan, Italy; 3 Department of Experimental Oncology, IEO, European Institute of Oncology IRCCS, Via Adamello 16, 20139 Milan, Italy; 4 Department of Medical Oncology, Urogenital and Head and Neck Tumors Medical Treatment, IEO, European Institute of Oncology IRCCS, Via Ripamonti 435, 20141 Milan, Italy; 5 Division of Radiotherapy, IEO, European Institute of Oncology IRCCS, Via Ripamonti 435, 20141 Milan, Italy; 6 Department of Oncology and Hemato-Oncology, School of Medicine, State University of Milan, Milan, Italy; 7 Division of Early Drug Development for Innovative Therapies, IEO, European Institute of Oncology IRCCS, Via Ripamonti 435, 20141 Milan, Italy

**Keywords:** Head and neck cancer, Lymphocytes, Tumour immunology

## Abstract

To investigate the role of the altered activation of the immune system in the prognosis of patients affected by laryngeal squamous cell carcinoma (LSCC). We analyzed 56 patients with advanced LSCC divided into two groups according to their prognosis: the first group relapsed within 24 months after treatment, the second group had no evidence of disease at 2 years. The presence of stromal tumor infiltrating lymphocytes (TILs) at the tumor-host border was investigated. In 43 patients we evaluated the expression of 395 genes related to immune system activation through a next generation sequencing panel. Priority-LASSO models and clustering analyses were integrated with multivariate Cox proportional hazard modeling to identify independent genes associated with relapse and estimate hazard ratios in relation to gene expression and TILs. TILs and the expression of genes related with immune system activation (FCGR1A, IFNA17, FCRLA, NCR3, KREMEN1, CD14, CD3G, CD19, CD20 and CD79A) were significantly associated with prognostic factors or disease specific survival. In patients with lymph node metastases and advanced T stage (pT4), the expression of other genes was altered. Low TILs count was highly associated with relapse within 2 years (p < 0.001). Low TILs and altered expression of specific genes associated with tumor-immune systems interactions emerged as independent risk factors, associated to poor prognosis and relapse within 2 years in advanced LSCC. Evaluation of patients’ immune profile could be useful for prognosis and future therapeutic approaches towards personalized therapy.

## Introduction

US population-based Surveillance, Epidemiology, and End Results (SEER) data indicate that the 5-year overall survival for patients with laryngeal cancer diagnosed in 2009–2015 was approximately 60%, with no improvement on survival over the period 1975 to 2012, notwithstanding advancement in both diagnosis and treatment^1,2^.

Just about less than half of patients with laryngeal cancer still present with locally advanced disease or regional nodal metastases, and in these patients 5-year overall survival is less than 50%^1,2^.

The treatment of advanced laryngeal carcinoma, stage III-IV, is challenging because of the need to combine effective local disease control with preservation of laryngeal function. Loss or compromise of laryngeal function has a major negative impact quality of life, with increased burden on family and society^3,4^. Current standard approaches to advanced laryngeal cancer are surgery with concurrent radiation (RT) or chemotherapy (CT), in accordance with tumor stage and patients’ clinical condition and wish^3^.

Nowadays immunomodulatory treatments have emerged as promising new approaches to advanced laryngeal and other head and neck cancers^5–7^.

Recent studies have characterized immune infiltrates in the tumor microenvironment (TME) of head and neck cancers and have suggested that low levels of stromal tumor infiltrating lymphocytes (TILs) are tightly associated with cancer relapses^8–10^. The quantity of the immune infiltrate is important to mount an efficient antitumor response and, in this regard, the transcriptional level of key genes involved in T cell and B cell function may reveal deregulation^11^.

Altered infiltrating immune cells in TME are associated with altered immune response activation, cancer progression and response to immunotherapy^12^. Immunotherapy can lead to cancer elimination neutralizing immunosuppressive checkpoints^12^. For instance, several effective immunotherapies using monoclonal antibodies targeting PD1 and its ligands PD-L1/2 enhance antitumor responses of TME infiltrating cells^12,13^. The immune infiltration of TME is also characterized by the expression of specific immune cell markers in cancer samples^13,14^.

Therefore, knowing the status of the TME and of the immune system, is fundamental for understanding the possible responses to new treatments.

The recurrence rates for laryngeal cancer range from 16 to 40%, despite advances in treatment^15^. Because of the high relapse rate in laryngeal cancers, biomarkers associated to prognosis and prediction of immunotherapy response would be highly desirable, also in light of the recent FDA approval of pembrolizumab as first-line treatment in patients with recurrent/metastatic head and neck squamous cell carcinoma^16^.

Our hypothesis is that an altered immune response activation leads to an ineffective tumor growth block, thus favoring the tumor escape phenomena and local relapse presentation.

In the present study we investigated a consecutive series of patients with advanced squamous cell laryngeal cancers who had standard treatment, with the aim of identifying immune-related factors correlating with outcomes. As immune factors, we investigated the expression of a large panel of genes (395) involved in immune response activation and the presence at the tumor-host border of stromal tumor-infiltrating lymphocytes (TILs). Our endpoints were: local relapse (LR) within 2 years of treatment in comparison to no evidence of disease (NED) at 2 years and disease- free survival (DFS).

## Materials and methods

### Study design

This was a single-center retrospective study approved by the Ethical Committee of the European Institute of Oncology (code IEO 662) on patients with advanced, pathological stage III or IV (TNM, AJCC, 8th Ed) squamous cell laryngeal carcinoma treated from 2008 to 2017 with curative intent at our Institute in accordance with NCCN guidelines^17^.

Eligibility criteria were: age over 18 years, no history of psychiatric disorder, no previous, synchronous or metachronous cancer, absence of infectious disease, absence of immune system alterations diagnosed at our Institute, at least 2 years of follow-up.

An informed consent informed consent was obtained for all the included patients.

Patients were divided into two groups based on their prognosis: those who had no evidence of disease at 2 years, NED, and those who relapsed within 24 months after treatment, LR.

Cases with cancer relapse showed no evidence of disease at 4 weeks after treatment either clinical or instrumental examination (such as magnetic resonance imaging, computed tomography, neck ultrasound or laryngeal endoscopy) according to the Response Evaluation Criteria in Solid Tumors (RECIST) criteria^18^. Patients with residual disease 4 weeks after treatment, were not included in the analysis. Thirty relapsed and 30 NED patients conforming to the eligibility criteria were identified.

Since tumor specimens were available only for 56 patients, TILs and gene expression evaluation could only be assessed in these 56 patients. Successful transcript analyses were obtained in 43/56 (76.8%) cases.

All data included information on age at diagnosis, sex, laryngeal sub-site, smoke and alcohol habits, clinical and pathological staging and treatment administered. Plasmatic neutrophil-to-lymphocyte ratio (NLR) were evaluated at the time of diagnosis and extracted from electronic clinical records.

### Tumor infiltrating lymphocytes (TILs)

For each of the 56 patients, we collected the tumor hematoxylin and eosin slides (H&E), stored in the archive of IEO pathology division. The presence of TILs was assessed in H&E section with an optic microscope with 20X10 ocular and X40 magnification. TILs were evaluated as a continue variable according to the guidelines reported by the “International TILs Working Group”^19–21^.

Two pathologists (FM & DL) evaluated a different field of the same slide through a double-blind system according to Salgado et al., 2015 and we averaged the two ratings^19^. Only the lymphocytes, plasma-cells and macrophages were counted, whereas necrotic cells, eosinophils and neutrophils were excluded. The TILs were studied in a 1 mm thick area selected independently by the two pathologists, at the edge of the tumor, including the tumor, the tumor margin and the stroma.

In two different single field X40 magnification, each pathologist evaluated the percentage of lympho-monocytic infiltration in the connective tissue, avoiding the lympho-monocytic hot-spot area and intra-tumor infiltration.

### RNA sequencing: oncomine immune response research assay

The RNA-based Next Generation Sequencing (NGS) panel Oncomine Immune Response Research Assay (OIRRA) (ThermoFisher, Waltham, MA, USA) was adopted to measure the expression of immune-related genes. This panel allowed the simultaneous evaluation of 395 genes related to immune system activation, such as genes associated with lymphocyte regulation, cytokine signaling, lymphocyte markers, checkpoint pathways and tumor characterization. In particular, lymphocytes markers defining through B cell lineage (CD19, CD20 or MS4A1, CD79 A-B, FOXP3), macrophage (CD68), T cell lineage (CD3, CD4, CD8), neutrophils lineage (CD66b) and NK cells (NCAM1 and NFKBIA) were included.

The RNA-sequencing analysis was performed on tumor area and on the surrounding non- neoplastic tissue, with a close connective interface comprehensive of TILs. In detail, six sections five-micrometer-thick were obtained from representative formalin-fixed paraffin-embedded (FFPE) tumor blocks and used for RNA extraction. The RNA was extracted automatically with the Promega Maxwell instrument (Promega, Madison, WI, USA) using the Promega Maxwell RSC RNA FFPE kit and was quantified with the Quantus fluorometer (Promega, Madison, WI, USA). According to manufacturer’s instruction, a step of RNA quality assessment using real-time PCR was performed. Afterwards, 10 ng of RNA were used for the library preparation and the subsequent chip loading, both automatically realized on the Ion Chef System (ThermoFisher, Waltham, MA, USA) and the sequencing step was run using the Ion S5 System (ThermoFisher, Waltham, MA, USA). The targeted RNA-sequencing analysis was obtained using the Torrent Suite ImmuneResponseRNA plugin that produced gene transcript data, as previously reported^22^.

### Statistical analysis

Patients’ characteristics are described in terms of median and interquartile range (IQR) for continuous variables and absolute and relative frequencies for categorical variables. The LR group and NED group were compared in terms of prognostic factors (age, pT, pN) and received treatment (p-value of Chi-square tests are presented).

RNA-sequencing data were obtained for the gene expression level evaluation. The statistical analysis was first based on univariate ANOVA test comparing LR and NED, considering a gene-expression fold change < -2 or > 2 and a p value < 0.05 (Fold change measures a change).

Results of priority-LASSO models and clustering analyses were integrated in multivariate Cox proportional hazard models, adjusting for prognostic and confounding factors, to identify genes independently associated with relapse free survival. Hazard ratios (HR) with 95% Confidence Intervals (95% CI) of the probability of relapse, from multivariate models, are presented. A heatmap was also generated by performing a sparse Partial Least Square-Differential Analysis (sPLS-DA) (tenfold cross-validation and 100 repeats) and selecting the most discriminative genes by using the first and second component loading vectors.

### Statistical insights

The Read Per Milion (RPM) data were log-transformed (after adding a pseudo-count of 1 to avoid non-finite values resulting from log2(0)).

Normalized, gene-level count data generated from the run, were further analyzed with Affymetrix Transcriptome Analysis Console (TAC) software. Results normalized by RPM were downloaded from Immune Response RNA plugin and then uploaded in the TAC software.

The statistical analysis was first based on univariate ANOVA test comparing LR and NED, considering a gene expression fold change < -2 or > 2 and a p value < 0.05.

A Volcano Plot was implemented in order to easily visualise the results of the differential expression analysis by adopting the package “EnhancedVolcano”^23^. Significant differentially expressed genes were evaluated according to the gene expression fold change and the p value obtained with univariate ANOVA.

Normal distribution of residuals of the full models was also checked. Given the high number of tests we adjusted P-values for multiple testing hypothesis (False Discovery Rate adjustment).

The second analysis was carried out considering as main endpoint time to relapse. Priority-LASSO Cox model (hierarchical approach) was applied to select genes able to predict the survival endpoint^23,24^. Disease-free survival (DFS) was defined as the interval in years, from the surgery to the first recurrence or last follow-up. We used priority-Lasso model with the following block structures, according to their level of priority. High priority was assigned to the clinical variables such as pN, pT and radiotherapy. A second priority was assigned to the gene expression data; finally, a low priority was assigned to the demographical variables such as age, sex and smoke. The lambda parameter, i.e. the regularization strength, was selected as the minimum value that maximizes the area under the specificity sensitivity curve.

A clustering analysis was computed using Spearman’s (r_s_) correlation, in order to identify genes able to represent most of the variability, but not closely correlated with each other. Hierarchical clustering was applied optimizing the best number of clusters by using the Calinski and Harabtz index (maximum value). The number of clusters was established adopting the package “NbClust”^25^. A sensitivity analysis was conducted to evaluate whether the cluster number previously chosen was the best one. Then a higher number of clusters was applied by visualizing the dendrogram of the genes. This choice allowed to better discriminate uncorrelated genes from each other.

A supervised classification model Partial Least Square–Differential Analysis (PLS-DA) was performed by using MixOmics Package^26^. In particular PLS-DA was evaluated by applying tenfold cross-validation with 100 repeats.

Multivariate Cox models were computed by applying a backward and forward selection to identify independent genes associated with time to relapse, adjusting for clinical variables (TNM, type of treatment) and socio-demographical variables (age, sex, smoking, alcohol). Only statistically significant variables were kept in the models. A collinearity analysis (VIF) was conducted on larger clusters to remove highly correlated genes. In addition, other genes were not kept because too sparse data: we studied boxplot distributions of LR and NED, Kaplan–Meier curves and Hazard Ratio’s estimates with 95th confidence intervals (unstable models were not considered). We also considered performances of the models indicated by Concordance Index (C-index) and chose the models with the highest performance index.

Gene expression levels were assessed both as continuous and categorical variables. A cut-off value of log of gene expression was chosen for each gene to present Kaplan–Meier curves and assessed by Log-rank test. Each chosen multivariate Cox model was presented by using gene expression as continuous and categorized variable and evaluating their role independently of other prognostic and confounding factors. We used median values in order to have a representative value of gene expression. Upper and lower quartiles were also investigated in order to verify whether the associations with relapse were more evident with extreme values. Logistic models were used to investigate the associations with prognostic factors categorized as binary variables (pT and pN) and low TILs (< 5%). For these clinical endpoints univariate and multivariate logistic models were computed and the performances of the different models were compared by calculating the area under the Receiver Operating Characteristics curve (AUROC). Odd ratios (ORs) with 95%CI, assessing the associations with lymph-node involvement, pT 4 vs pT2-3 and low TILs, obtained from chosen multivariate models, are presented. Cohen Kappa and 95% CI for the agreement between TILs evaluations by the two pathologists are reported. In order to investigate the association with time to relapse, we considered a mean value of the evaluations of TILs assessed by the two pathologists. The cut-off point was chosen based on “International TILs Working Group” guideline^19,20^.

## Results

Of 56 patients, median age was 63 years, 86% (n = 48) were male, 54% (n = 30) were smokers, 87% (n = 49) were pT3-4 and 53% (n = 29) had lymph node metastasis (Table 1).

**Table 1:** Characteristics of the 56 patients and their cancers.

Characteristic	Category	N = 56 (100)
Age, Median (IQR)		63 (55–71)
Sex, n (%)	Male	48 (86)
	Female	8 (14)
BMI, Median (IQR), Kg/m^2^		25 (23.5–27.4)
Alcohol, n. (%)	Yes	4 (7)
	No	52 (93)
Smoker, n. (%)	Yes	26 (46)
	No	30 (54)
Side, n. (%)	Right	16 (28)
	Left	21 (38)
	Median	11 (20)
	Bilateral	8 (14)
Disease site, n. (%)	Glottic	21 (37)
	Hypoglottic	2 (4)
	Transglottic	7 (13)
	Sovraglottic	26 (46)
pT, n. (%)	1	2 (4)
	2	5 (9)
	3	23 (41)
	4	26 (46)
pN, n. (%)	0	26 (47)
	1	7 (13)
	2a	2 (4)
	2b	10 (18)
	2c	7 (13)
	3	3 (5)
Leukocytes, Median (IQR)		8.15 (6.5–9.8)
Neutrophils, Median (IQR)		5 (4.1–6.3)
Eosinophils, Median (IQR)		0.17 (0.08–0.26)
Basophils, Median (IQR)		0.03 (0.01–0.04)
Lymphocytes, Median (IQR)		1.88 (1.5–2.4)
Monocytes, Median (IQR)		0.7 (0.5–0.8)
Neutrophil-to-lymphocyte ratio (relative count) Median (IQR) ABBREVIATIONS MUST BE DEFINED		2.33 (1.97–3.17)
Neutrophil-to-lymphocyte ratio (absolute count) Median (IQR)		2.43 (2.02–3.43)
Treatment, n (%)	Surgery + RT + CT	16 (29)
	Surgery + RT	30 (53)
	Surgery + CT	1 (2)
	Surgery	8 (14)
	RT	1 (2)

*RT* radiotherapy; *CT* chemiotherapy.

After surgery was performed on 55 patients, 84% (n = 47) underwent adjuvant treatment: 53% (n = 30) RT, while 29% (n = 16) RT + CT. Only 16% (9 cases) had a single mode treatment (Table1).

Thirty LR patients and 30 NED patients were enrolled, but adequate tumoral specimen was available only for 56 patients to evaluate TILs and only 43 specimens were suitable for gene expression analyses.

Patients with LR and NED were compared for prognostic factors and treatments. No statistically significant difference was found between the groups in which the OIRRA analyses was possible (Table 2).

**Table 2:** Prognostic factors and treatment of 43 patients studied through the OIRRA panel.

	NED	Local Relapse	All	P-value^¥^
n. (%)	24 (100)	19 (100)	43 (100)	
Age*				
< 62	9 (37.5)	10 (52.6)	19 (44.2)	0.32
≥ 62	15 (62.5)	9 (47.4)	24 (55.8)	
**pT**				
1–3	12 (50.0)	9 (47.4)	21 (48.8)	0.86
4	12 (50.0)	10 (52.6)	22 (51.2)	
**pN**				
0	9 (37.5)	8 (42.1)	17 (39.5)	0.76
+	15 (62.5)	11 (57.9)	26 (60.5)	

*Median value of the evaluated sample.

^¥^Chi-Square test.

### RNA-sequencing analysis and gene expression comparison between patients with or without a relapse

We obtained successful transcript analyses in 43 of the 56 cases (76.8%) (19 LR and 24 NED). The sequencing results of the other 13 cases did not achieve the quality established for a robust gene expression evaluation (mapped reads > 1 million and valid reads > 80%).

ANOVA analysis showed that the expression of 22 genes differed significantly between the LR and NED groups. In detail, 3 genes (HLA-DQB2, HLA-A and GATA3) were up-regulated and 19 genes (CD53, IL23A, NOS2, NCR1, FCRLA, FCGR2B, TNFSF18, KLRF1, CD79A, LRG1, JCHAIN, IRF4, IFNA17, FCGR3B, CD79B, CTAG1B, FCGR1A, MADCAM1 and TNFRSF17) were down-regulated in the LR group, as reported in Table 3.

**Table 3:** Genes differentially expressed between patients who relapsed within two years (LR) and those with no evidence of disease at two years (NED).

	NCBI name	Gene function	Fold change of LR vs NED	P-value
CD53*	CD53 Molecule Absolute Nonsese	Adhesion, migration	− 6.9	0.001
IL23A*	Interleukin 23 Subunit Alpha	Dendritic cell, macrophage	− 3.5	0.016
NOS2*	Nitric Oxide Synthase 2	Innate IR	− 3.5	0.049
NCR1*	Natural Cytotoxicity Triggering Receptor 1	NK cell marker	− 3.0	0.026
FCRLA*	Fc Receptor Like A	B cell marker	− 3.6	0.014
FCGR2B*	Fc Fragment Of IgG Receptor IIb	B cell marker	− 4.6	0.002
TNFSF18*	TNF Superfamily Member 18	Checkpoint pathway	− 5.4	0.014
KLRF1*	Killer Cell Lectin Like Receptor F1	NK activation	− 2.9	0.015
CD79A*	CD79a Molecule	B cell receptor signalling	− 7.3	0.002
LRG1*	Leucine Rich Alpha-2-Glycoprotein 1	Neutrophil	− 5.8	0.001
JCHAIN*	Joining Chain Of Multimeric IgA And IgM	B cell marker	− 2.4	0.019
IRF4*	Interferon Regulatory Factor 4	Interferon signalling	− 3.8	0.014
IFNA17*	Interferon Alpha 17	T cell receptor signalling	− 3.0	0.005
FCGR3B*	Fc Fragment Of IgG Receptor IIIb	NK activation	− 2.0	0.012
CD79B	CD79B Molecule	B cell receptor signalling	− 3.3	0.032
CTAG1B	Cancer Testis Antigen 1B	Tumor antigen	− 4.2	0.014
FCGR1A	Fc Fragment of IgG Receptor Ia	B cell marker	− 3.2	0.007
MADCAM1	Mucosal Vascular Adressin Cell Adhesion Molecule 1	Adhesion, migration	− 2.2	0.025
TNFRSF17	Tumor Necrosis Factor Receptor Superfamily Member 17	B cell marker	− 4.2	0.018
HLA-DQB2*	Major Histocompatibility Complex, Class II, DQ Beta 2	Antigen processing	2.17	0.032
HLA-A*	Major Histocompatibility Complex, Class I, A	Antigen processing	14.62	0.013
GATA3	GATA Binding protein 3	Helper T cell	2.2	0.045

P-values from univariate ANOVA model.

*Differentially expressed genes between local relapse (LR) and no evidence of disease (NED) groups, according to Transcriptome Analysis Console (TAC) software analysis.

**Figure 1:**
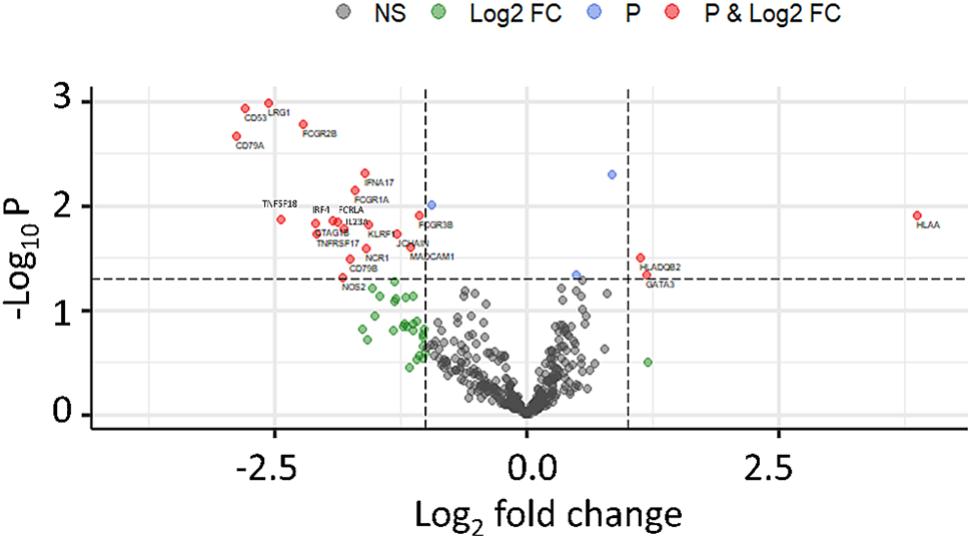
Volcano plot of Log_2_ fold change of differential expression analysis for local relapse (LR) and no evidence of disease (NED). Red points represent genes with gene-level fold change (< -2 and > 2) and p value (< 0.05). Green and blue points indicate genes with fold change or p-value that respect only one of the two cut off values, fold change and P-values respectively. Grey points are no significant genes.

A volcano plot confirmed these results, showing the presence of transcripts highly representative of LR or NED groups in terms of fold changes and p values (from ANOVA) (Fig. 1).

### Correlations between immune-related gene expression and TILs with disease free survival and prognostic factors

**Figure 2:**
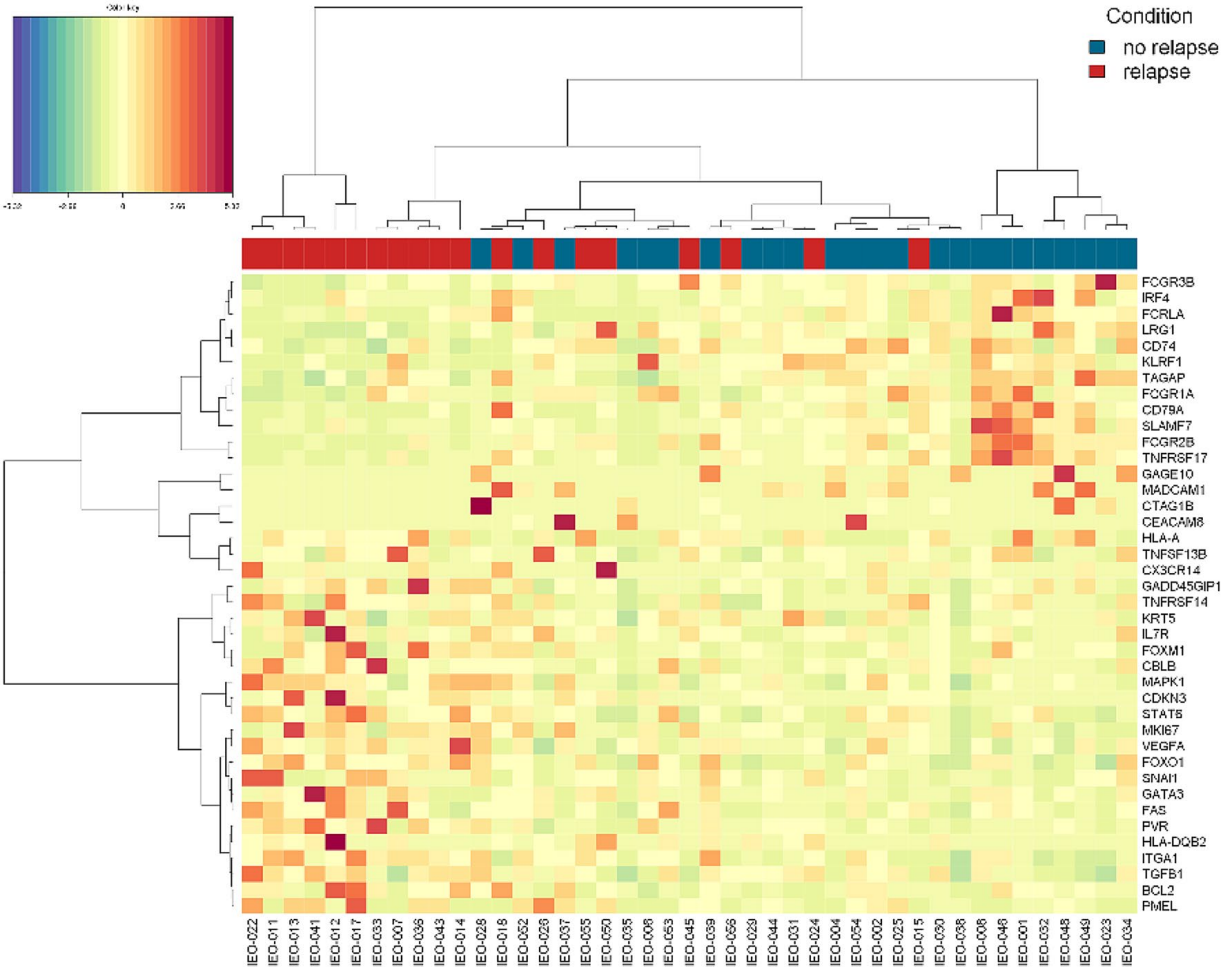
Heatmap of the genes studied for patients with local relapse (LR) and no evidence of disease (NED).

**Figure 3:**
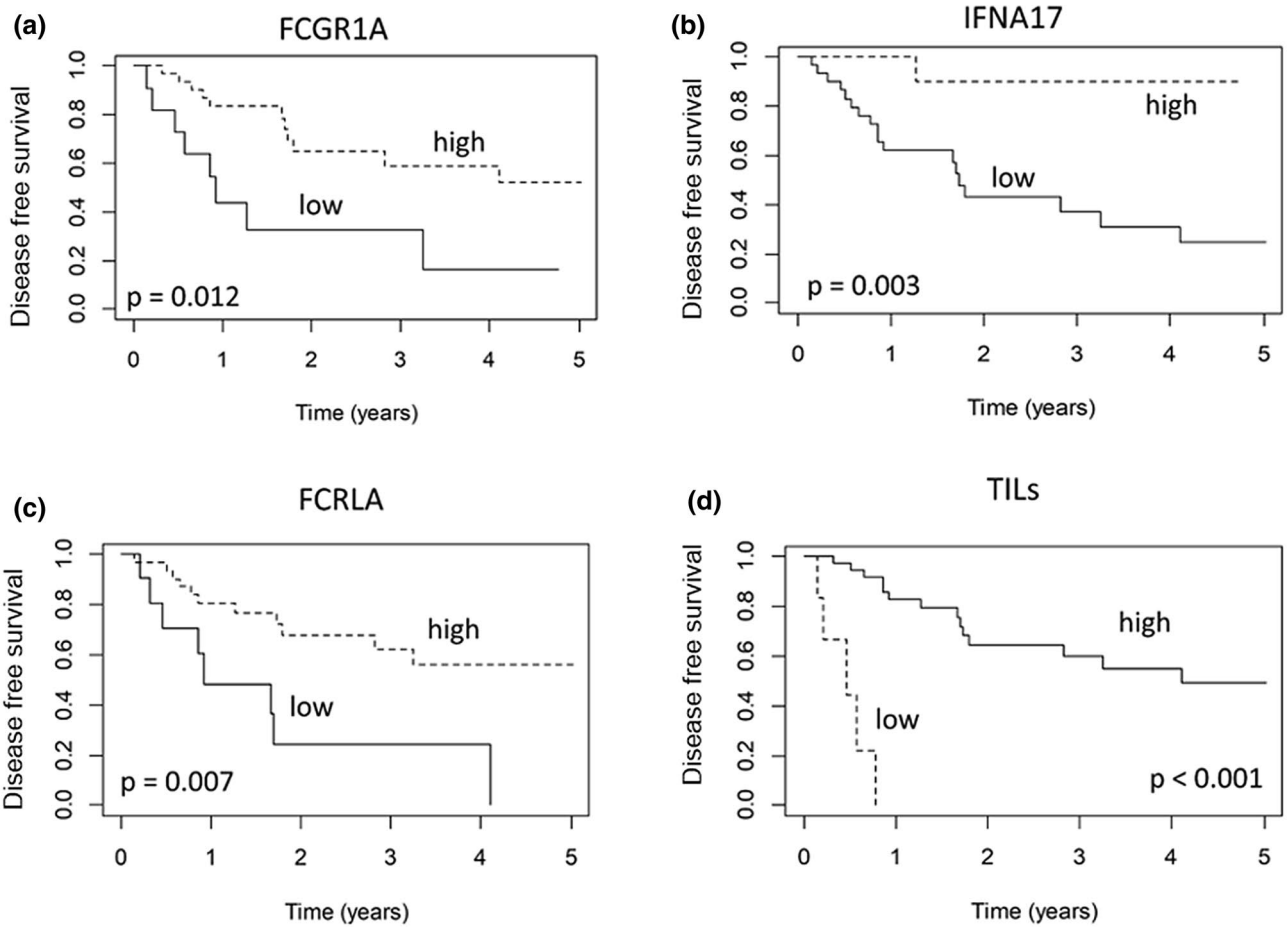
**(a–d)** Disease free survival according to gene expression of FCGR1A (a), IFNA17 (b) and FCRLA (3c) and TILs evaluation (3d).

**Figure 4:**
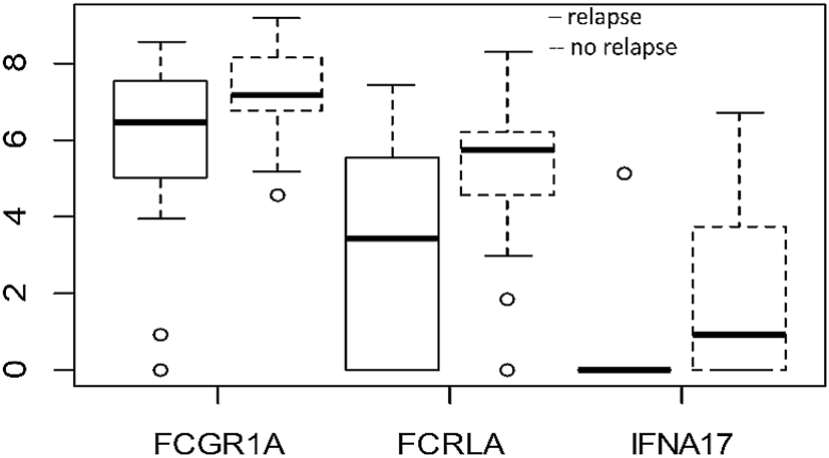
Box plot of log expression of genes (FCGR1A, IFNA17 and FCRLA) found to be significantly associated with the local relapse in Cox multivariate models.

As displayed by the heatmap in Fig. 2, specific gene expression profiles distinguished the LR and NED groups. Evaluating the association between gene expression and DFS, we identified three genes significantly associated with relapse, namely FCGR1A, IFNA17 and FCRLA. The associations of these genes with relapse were evaluated in the multivariate Cox models after applying a feature selection with a priority-Lasso. A down-regulation of these three genes were significantly associated to a greater risk of relapse, as shown in Kaplan–Meier curves of RFS and log-rank tests in Figs. 3 and 4.

**Figure 5:**
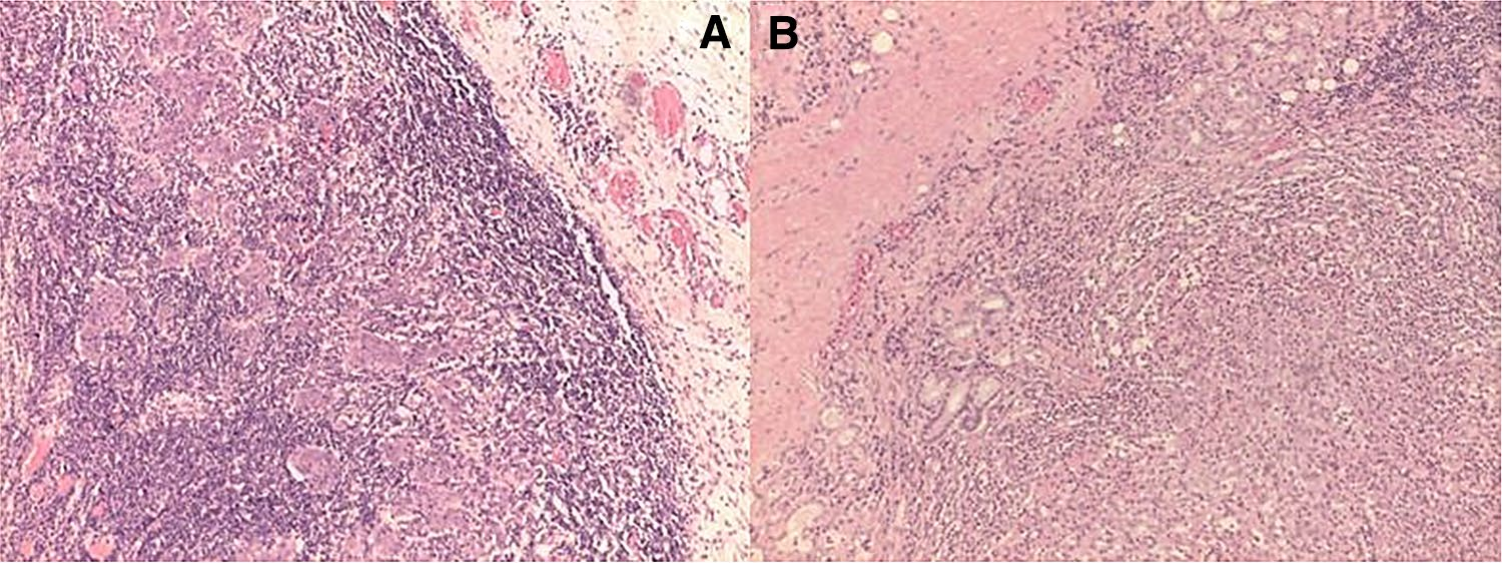
(**A**,**B**) Hematoxylin and eosin stained slides of LSCC cases characterized by high (> 5%) (**A**); or low (< 5%) TILs (**A**).

We evaluated the association between the presence of TILs and prognosis. The double-blind system used to evaluate TILs showed a good agreement between pathologists (Cohen Kappa = 0.69, 95% CI: 0.50–0.87). Therefore, in order to investigate the association with time to relapse, we considered a mean value of the evaluation of the two pathologists. In the 43 specimens analyzed with OIRRA, TILs were < 5% in 6 patients and > 5% in 37, with a median value of 30.5 (IQR: 14.25–55, absolute range: 1–85). We found that patients with stromal TILs < 5% had a significantly greater risk of relapse (p < 0.001) (Figs. 3d, 5A,B).

**Figure 6:**
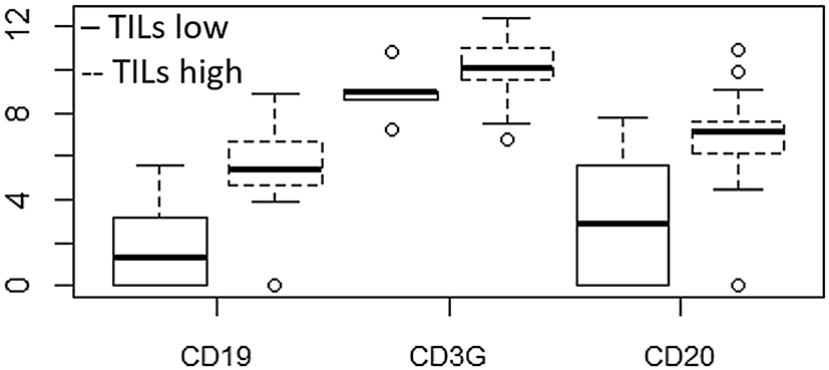
Box plot of log expression of genes (CD19, CD3G, CD20) found to be significantly associated with high levels of TILs.

Among the lymphocyte markers evaluated through the OIRRA panel, we found that an upregulation of CD19, CD20 and CD3G was significantly associated with high-TILs value (p = 0.02, 0.03, 0.04 respectively in univariate analysis), whereas CD8A showed a positive correlation trend (p = 0.07). No other significant association with TILs levels was identified (Table 4, Fig. 6).

**Table 4:** Results of univariate logistic models: genes associated with Tumor infiltrating lymphocytes (TILs).

Gene	Odd Ratio	Low 95CI	Up 95CI	p-value
CD19	0.65	0.46	0.93	0.02
CD3D	0.80	0.54	1.17	0.25
CD3E	0.77	0.51	1.15	0.20
CD3G	0.46	0.22	0.95	0.04
CD4	1.02	0.58	1.78	0.96
CD40	0.47	0.16	1.40	0.17
CD40LG	0.99	0.71	1.37	0.94
CD44	0.86	0.45	1.67	0.66
CD68	2.01	0.49	8.31	0.33
CD79A	0.83	0.66	1.05	0.11
CD79B	0.86	0.64	1.15	0.32
CD8A	0.63	0.39	1.04	0.07
CD8B	0.79	0.57	1.11	0.17
CD66b	0.86	0.38	1.92	0.71
FOXP3	0.80	0.60	1.08	0.15
NCAM1	1.31	0.86	1.99	0.21
NFKBIA	0.82	0.32	2.07	0.67
CD20	0.75	0.57	0.97	0.03

*Odd ratio* indicates the association of gene expression with TILs < 5%.

The prognostic role of TILs was confirmed in the multivariable analysis. Indeed, even including the stromal TILs evaluation in the multivariate Cox model analysis and considering the gene expression value as a dichotomic variant, the presence of TILs and high expressions of FCGR1A, IFNA17 and FCRLA remained independent factors significantly associated with a good prognosis (Table 5).

**Table 5:** Results from Multivariate Cox models: Hazard ratios with 95% Confidence intervals assessing the association with time to local relapse (LR).

Variables	Contrasts	Hazard ratio	Low .95 CI	Up .95 CI	P-value
**I model**					
FCGR1A	High vs low	0.21	0.08	0.56	0.001
FCRLA	High vs low	0.34	0.13	0.89	0.02
IFNA17	Positive vs null	0.06	0.009	0.53	0.01
**II model**					
FCGR1A	High vs low	0.30	0.10	0.90	0.03
FCRLA	High vs low	0.32	0.12	0.85	0.02
IFNA17	Positive vs null	0.09	0.01	0.74	0.02
TILs stromal	≥ 5% vs < 5%	0.06	0.12	0.38	0.002

Cut-off identified considering quantiles of log gene expression; High (n = 33) means greater than first quartile (n = 11), for IFNA17 the first quartile is zero; TILs: n = 37 with ≥ 5 and n = 6 with < 5.

**Figure 7:**
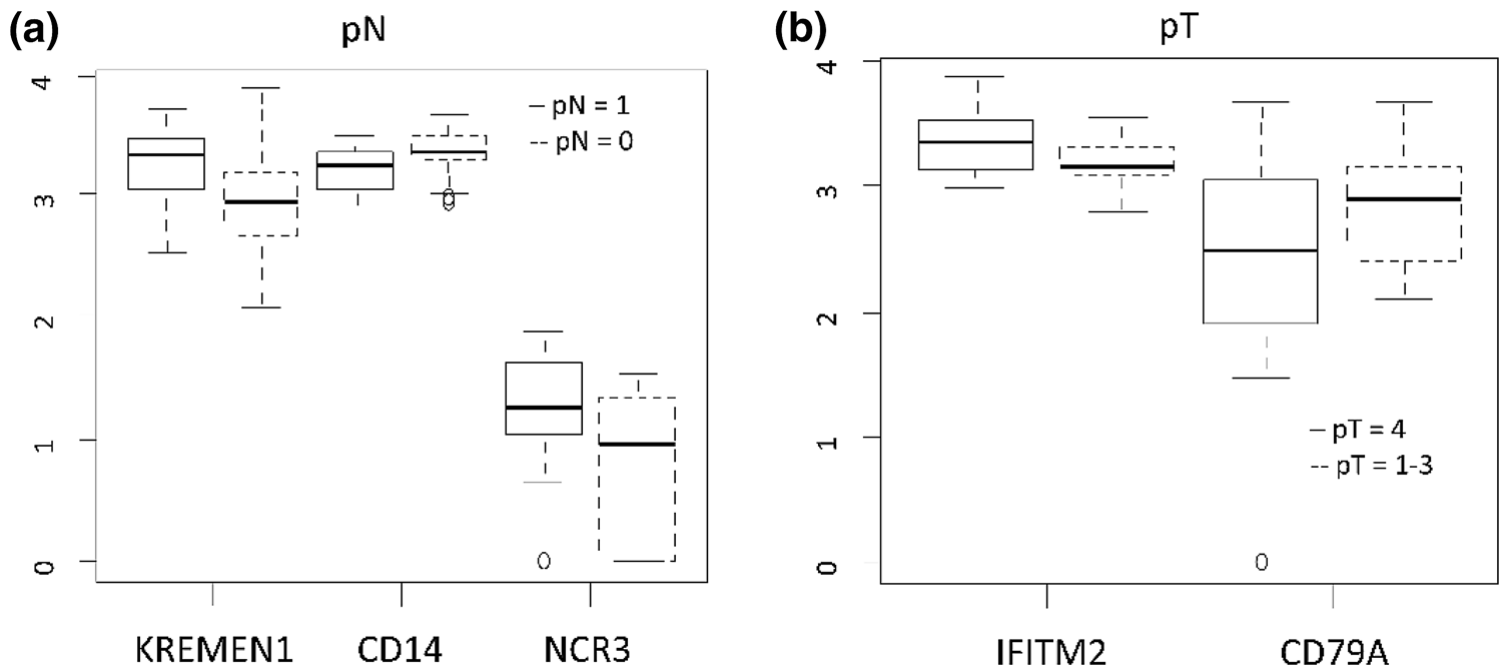
(**a**, **b**) Box plot of log expression of genes associated with Lymph-nodes status (pN) (6a) and Tumor staging (pT) (6b).

The multivariate logistic models showed that the expression of three genes were independently associated with metastatic lymph-nodes (pN = 0 vs pN +): KREMEN1, CD14 and NCR3. KREMEN1 and NCR3 are up-regulated and CD14 is down-regulated (Table 6, Fig. 7).

**Table 6:** Results of multivariate logistic models: Odd ratios assess the association of gene expression with pN status and pT status.

Endpoint	Variable	Contrasts	Odd Ratio	Low.95 CI	Up.95 CI	P-value
pN: pN + vs pN0	KREMEN1	High vs low	10.98	1.66	72.57	0.01
	CD14	High vs low	0.07	0.009	0.55	0.01
	NCR3	High vs low	26.15	2.21	308.73	0.009
pT: pT4 vs pT1-3	IFITM2	High vs low	7.1	1.21	41.56	0.02
	CD79A	High vs low	0.26	0.06	1.02	0.05

KREMEN1 (Kringle Containing Transmembrane Protein 1); CD14 (CD14 Molecule); NCR3 (Natural Cytotoxicity Triggering Receptor 3); IFITM2 (Interferon Induced Transmembrane Protein 2); CD79A (CD79a Molecule); Q: quartile; Cut-off refers to quantiles of log gene expression. For Kremen1, CD14 and CD79A ‘high’ refers to greater than median value, for NCR3 ‘high’ refers to greater than first quartile (n = 11) and for IFITM2 greater than third quartile (n = 11).

Two genes were found to be independently associated with tumor size (pT4 vs pT1-3): IFITM2 and CD79A. IFITM2 was up-regulated and CD79A down-regulated in pT4. Finally, C-index of multivariate logistic model assessing the association with pT was 0.67, indicating a good performance of this model (Table 6, Fig. 7).

Furthermore, high expression of CD19, CD79A, CD79B and CD20 were associated with a significantly better prognosis (p = 0.002, 0.003, 0.04, 0.01 respectively in univariate analysis). Spearman's correlation between the FCRLA, CD40 and CD20 genes confirmed the good correlation between CD20 and FCRLA (r_s_ = 0.58).

We did not find any correlation of expression levels of PD-L1, PD-1 and CD40 between relapse and the expression of all factors included in the OIRRA RNA-sequencing panel. No association between the neutrophil to lymphocyte ratio and prognostic features was observed (data not shown).

## Discussion and conclusions

In this study, we investigated the role of immune system activation in advanced LSCC. We analyzed two cohorts of patients with very different prognosis, divided into local relapse (LR) or no evidence of disease (NED) groups. In the study cohort low levels of TILs and altered expression of immune system activation related genes are tightly associated with LR.

In advanced LSCC we highlighted and confirmed the role of TILs as an independent prognostic factor^8,9^. Recently, different studies underline the role of the immune cell infiltrates in head and neck cancer. The quality and quantity of TILs determines the antitumor response, being directly related with patients’ prognosis and DFS^10^. These data are reported not only for laryngeal cancer but also for HPV-positive oropharyngeal and tongue squamous cell carcinoma^8–10,27^.

TILs help the immune system to achieve disease control and tumor healing. Indeed, the quantity of the immune infiltrate is important to mount an efficient antitumor response and, in this regard, the transcriptional level of key genes involved in T cell function may reveal deregulation in the immune system^28^. TILs are a histopathological well-established prognostic parameter for several types of tumors and have been included in an integrated grading staging system for head and neck squamous cell carcinoma^27–31^.

Our data suggest a significant correlation between high-level of TILs which enrich the tumor-host interface and both B-cell lineage (CD20 and CD19) and T-cell lineage (CD3G and CD8) (Table 4, Fig. 6). The association with these genes (CD19, CD20, CD3G and CD8A) could increase the response to treatment and DFS, as observed in breast, ovarian and renal cell carcinoma^32–35^. The interaction via cytokine secretion by CD19 and CD20 is followed by the activation and up-regulation of CD8A, leading to a cytolytic effect which increases tumor control and therapy response^32^. Of the other genes analyzed, we did not find any significant association with TILs and DFS (Table 4, Fig. 6).

Moreover, by multivariate analyses, adjusting for all known prognostic factors and tumor characteristics, of the 19 down regulated genes emerged with OIRRA, only three (FCGR1A, FCRLA and IFNA17) were identified as independent risk factors for DFS, upon comparing the average transcript gene expression in 19 LR patients with those in 24 NED patients.

FCGR1A and FCRLA encode for proteins similar to the Fc fragment of γ immunoglobulin. FCRLA is a soluble resident protein of the endoplasmic reticulum of B cells which binds intracellular immunoglobulins expressed in B-cell lineage (CD19 and CD20)^36–39^. The expression of FCRLA was recently discovered in human immature plasmacytoid dendritic cells (pDCs), indicating that FCRLA could perhaps participate in chaperone-mediated protein folding both in pDCs and B cells^39^. In our series, CD40 gene related with pDC was not associated with TILs. On the contrary, CD19 and CD20, B cell lineage clusters of differentiation related genes, showed a high association with high-TILs and CD19 also with DFS (p = 0.002), as CD79A and CD79B (p = 0.003 and p = 0.04 respectively). CD20 showed an association with FCRLA, thus suggesting a role in tumor antigen presentation following an immune-mediated pathway directly on pDCs.

These mature cells stimulate the immune system for the antigen presentation process to T-cell subset to achieve the elimination of neoplastic cells^39^.

FCRLA is expressed in B lineage lymphoma and its up-regulation was associated with a good response in patients treated with rituximab, an anti-CD20 immunotherapy drug^40^.

In our study CD20 was associated with prognosis (p = 0.011) and the correlation with FCRLA was good (r_s_ > 0.5). A down-regulation of FCRL genes family confers poor prognosis in chronic lymphocytic leukemia^41^, as in our cases. In the studied cohort, none of the patients were treated with immunotherapy regimens, thus we could not speculate on any predictive role of FCRLA towards therapy response.

FCGR1A has a pivotal role in chronic inflammatory diseases and in response to infections^42,43^. Moreover, FCGR1 protein activates the phagocytic activity of myeloid-cells, inducing antitumoral activity^44,45^. Interestingly, the binding receptor, the Fc fragment of γ immunoglobulin, could also influence the anti-tumor activity of antibodies against immune checkpoint targets^46^.

Of note, head and neck squamous cell carcinoma is infiltrated by B cells (CD19, CD20) producing immunoglobulins and immature pDCs that can produce type I interferons (IFNs), including IFN-α, although why the association to CD19 with FCRLA or CD20 with FCRLA up-regulate the IFN-α, through pDCs, is not clear^39,47,48^. However, the type I IFNs could have significant antitumor efficacy, beyond the activation of the host innate immune response^49–51^.

As reported by R.A. Ningrum (2014), IFN-α acts directly against tumor growth by inhibiting the cancer cell proliferative activity, or indirectly by activating the cytotoxic T-cells and NK cells and increasing cytokine secretion^52^. The therapy with type I interferons, including IFN-α alone or in association with other drugs, is useful in many types of malignant tumors such as melanoma, hairy cell leukemia, renal cell carcinoma, increasing the DFS^52^. In our analysis we found that the expression of IFNA17, a type I interferon, was up-regulated in patients with better prognosis.

All the patients included in this study were affected by advanced laryngeal cancer for tumor stage (pT) or lymph-node stage (pN).

As reported in the NCCN guidelines the advanced stage of laryngeal cancer (stage III-IV) is defined according to the lymph node status (pN) and the tumor status (pT).

pT is further defined as a moderately or very advanced tumor when it invades through the external cortex of the thyroid cartilage and / or invades the tissues beyond the larynx (pT4) and / or in the presence of lymph node metastases (pN +)^17^. Following this definition, we found genes differentially expressed associated to the presence of metastatic lymph-node (pN + vs pN0) or advanced pathological T stage (pT4 vs pT1-3).

KREMEN1, and NCR3 were up-regulated and significantly linked to the presence of lymph-nodal metastasis (pN +); CD14 was down-regulated.

KREMEN1 is a transmembrane receptor protein that functionally cooperates with Dickkopf-1 (DKK1) to block wingless (WNT)/β -catenin signaling, the activation of which leads to increased apoptosis in melanoma cells^53^. KREMEN1 is connected with the (WNT)/β -catenin pathway which facilitates apoptosis in melanoma cells. If KREMEN1 is up regulated, cell apoptosis is avoided and tumor growth is facilitated as well as metastases, as in our cases.

NCR3 encodes for a natural cytotoxicity receptor (NCR), which is a trans-membrane receptor with 1–2 extracellular domain and expressed exclusively in NK-cells, playing a role in triggering NK-mediated tumor cells killing^54^. An upregulation of NCR3 can reduce the immature pDC activity, reducing the type I IFN and increasing the tumor metastatic potential, as reported in metastatic gastrointestinal stromal tumor^55^, melanoma metastasis^56^ and as in our cases (Fig. 7a).

This effect is in contrast with what we observed for IFN-α: high level of IFN-α was linked to an increase of immature pDCs activity and better prognosis. Instead, a high-expression of NCR3 caused an increased NK-mediated cells killing, with immature pDCs lysis increasing the cancer metastatic potential^55,57^.

We observed a down-regulation of CD14 in lymph node positive cases. CD14 encodes for a surface antigen, preferentially expressed on monocytes/macrophages myeloid derived suppressor cells (MDSC) that mediates the innate immune response to bacterial lipopolysaccharide^58^. In high risk neuroblastoma patients, low expression of CD14 was reported to correlate with tumor advanced stage and with lymph-node metastasis^59^.

Moreover, we noted an up-regulation of IFITM2 and a downregulation of CD79A in patients with pT4 tumor (Fig. 7b). IFITM2 encodes for interferon induced transmembrane protein 2 and is associated to cancer growth and metastasis in gastric cancer, whereas the expression of CD79A, a B lymphocytes antigen receptor, is a good prognostic factor in hepatocellular carcinoma^60,61^.

All these significant alterations in factors controlling the host immune response raise the hypothesis that an altered immune response activation leads to an ineffective tumor growth block, thus favoring the tumor escape phenomena and local relapse.

To date, patients affected by advanced LSCC are treated with surgery, as in our Institute, and standard radio-chemotherapy regimens, but the prognosis remains poor^62,63^. Biomarkers associated to prognosis or predictive of therapy response are still lacking in LSCC. An improvement in the survival could be reached with the forthcoming introduction of new immunotherapy regimens for the first-line treatment of patients with metastatic or unresectable recurrent head and neck squamous cell carcinoma. But it is still to be defined how immune system activation can boost standard chemotherapy and radiotherapy regimens towards tumor control.

Molecular and cellular changes which occur at strategic points in the control of tumor spread, such as at the level of immune checkpoints can lead to an altered, reduced or even absent therapeutic response if patients with these alterations are treated with standard therapies. The association with immunotherapy can instead overcome this issue and implement the therapeutic response^64^.

This study has potential limitations, such as the retrospective nature of the analysis, the absence of automated TILs analysis on the entire tumor region with specific software tools to avoid possible subjective interpretations of the results and the very limited number of patients. A larger cohort of patients should validate these preliminary results.

Despite this, to the best of our knowledge, this is the first report correlating TILs with a gene signature in advanced laryngeal cancer relapses and could be considered a proof of concept to address further studies. The morphological analysis of TILs could be routinely performed for the diagnosis of head and neck cancer as they are important predictive parameters, cheap and easy to evaluate after a short training.

The patients’ gene-signature study could be used, in the future, for choosing the best therapy according to patients’ characteristics, as the complex interaction between cancer and the host immune system is the key for future personalized treatments of patients affected by head and neck cancer. Indeed, heading to tailored therapy, an evaluation of the immune profile could be useful both for prognostic and therapeutic purposes, since immunotherapy regimens are becoming part of the personalized medicine for head and neck cancer patients.
