# Author Correction: A role for the immune system in advanced laryngeal cancer

**DOI:** 10.1038/s41598-021-89174-8

**Published:** 2021-05-03

**Authors:** Marta Tagliabue, Fausto Maffini, Caterina Fumagalli, Sara Gandini, Daniela Lepanto, Federica Corso, Salvatore Cacciola, Alberto Ranghiero, Alessandra Rappa, Davide Vacirca, Maria Cossu Rocca, Daniela Alterio, Elena Guerini Rocco, Augusto Cattaneo, Francesco Chu, Stefano Zorzi, Giuseppe Curigliano, Susanna Chiocca, Massimo Barberis, Giuseppe Viale, Mohssen Ansarin

**Affiliations:** 1 Division of Otolaryngology and Head and Neck Surgery, IEO, European Institute of Oncology, IRCCS, Via Ripamonti 435, 20141 Milan, Italy; 2 Division of Pathology, IEO, European Institute of Oncology IRCCS, Via Ripamonti 435, 20141 Milan, Italy; 3 Department of Experimental Oncology, IEO, European Institute of Oncology IRCCS, Via Adamello 16, 20139 Milan, Italy; 4 Department of Medical Oncology, Urogenital and Head and Neck Tumors Medical Treatment, IEO, European Institute of Oncology IRCCS, Via Ripamonti 435, 20141 Milan, Italy; 5 Division of Radiotherapy, IEO, European Institute of Oncology IRCCS, Via Ripamonti 435, 20141 Milan, Italy; 6 Department of Oncology and Hemato‑Oncology, School of Medicine, State University of Milan, Milan, Italy; 7 Division of Early Drug Development for Innovative Therapies, IEO, European Institute of Oncology IRCCS, Via Ripamonti 435, 20141 Milan, Italy

Correction to: *Scientific Reports* 10.1038/s41598-020-73747-0, published online 27 October 2020

This Article contains an error in the entry Neutrophil-to-lymphocyte ratio (relative count) Median (IQR) reported in Table 1.

“Neutrophil-to-lymphocyte ratio (relative count) Median (IQR) ABBREVIATIONS MUST BE DEFINED”.

should read:

“Neutrophil-to-lymphocyte ratio (relative count) Median (IQR)”.

Secondly, the caption of Table 1 contains an error.

“Table 1. Characteristics of the 56 patients and their cancers. *RT* radiotherapy; *CT* chemiotherapy.”

should read:

“Table 1. Characteristics of the 56 patients and their cancers. *RT* radiotherapy; *CT* chemiotherapy; *IQR* interquartile range.”

